# Therapeutic intervention in neuroinflammation for neovascular ocular diseases through targeting the cGAS-STING-necroptosis pathway

**DOI:** 10.1186/s12974-024-03155-y

**Published:** 2024-06-25

**Authors:** Biyan Ni, Ziqi Yang, Tian Zhou, Hong Zhou, Yang Zhou, Shiya Lin, Huiyi Xu, Xiaojing Lin, Wei Yi, Chang He, Xialin Liu

**Affiliations:** 1 State Key Laboratory of Ophthalmology, Zhongshan Ophthalmic Center, Sun Yat-sen University, Guangzhou, 510060 China; 2 Guangdong Provincial Key Laboratory of Ophthalmology and Visual Science, Guangzhou, 510060 China

**Keywords:** Pathological angiogenesis, STING, Myeloid cells, Necroptosis, Neuroinflammation, Immunotherapy, Neovascular ocular diseases

## Abstract

The microglia-mediated neuroinflammation have been shown to play a crucial role in the ocular pathological angiogenesis process, but specific immunotherapies for neovascular ocular diseases are still lacking. This study proposed that targeting GMP-AMP synthase (cGAS)-stimulator of interferon genes (STING) might be a novel immunotherapy for these angiogenesis diseases. We found a significant upregulation of *CGAS* and *STING* genes in the RNA-seq data derived from retinal tissues of the patients with proliferative diabetic retinopathy. In experimental models of ocular angiogenesis including laser-induced choroidal neovascularization (CNV) and oxygen-induced retinopathy (OIR), the cGAS-STING pathway was activated as angiogenesis progressed. Either genetic deletion or pharmacological inhibition of STING resulted in a remarkable suppression of neovascularization in both models. Furthermore, cGAS-STING signaling was specifically activated in myeloid cells, triggering the subsequent RIP1-RIP3-MLKL pathway activation and leading to necroptosis-mediated inflammation. Notably, targeted inhibition of the cGAS-STING pathway with C-176 or SN-011 could significantly suppress pathological angiogenesis in CNV and OIR. Additionally, the combination of C-176 or SN-011 with anti-VEGF therapy led to least angiogenesis, markedly enhancing the anti-angiogenic effectiveness. Together, our findings provide compelling evidence for the importance of the cGAS-STING-necroptosis axis in pathological angiogenesis, highlighting its potential as a promising immunotherapeutic target for treating neovascular ocular diseases.

**Supplementary Information:**

The online version contains supplementary material available at 10.1186/s12974-024-03155-y.

## Introduction

Pathological angiogenesis is a prevalent feature in various human neovascular ocular diseases, including diabetic retinopathy (DR), neovascular age-related macular diseases (nAMD), etc [1]. During this pathological process, the myeloid cells including microglia and infiltrating monocyte-derived macrophages are activated, inducing neuroinflammation in the retina and choroid. These immune cells release pro-inflammatory factors and other molecules that stimulate endothelial cell proliferation and migration, consequently leading to the formation of abnormal neovessels [2]. Steroids, employed as non-specific anti-inflammatory therapies, have been widely used to treat neovascular ocular diseases [3]. However, their efficacy is limited with risks of potential side effects [4]. Therefore, mechanistic investigations into the immune-inflammatory pathways involved in angiogenesis and the identification of potential specific immunotherapeutic targets are highly warranted.

The cGAS-STING pathway has recently emerged as a critical innate immune signaling mechanism in host defense. cGAS primarily recognizes cytosolic DNA, particularly pathogenic DNA from invading viruses and bacteria, and triggers downstream IRF3/IRF7 or NF-κB signaling, resulting in the production of type I interferons and other inflammatory cytokines [5, 6]. Unraveling the potential implications of the cGAS-STING pathway beyond infection could offer valuable insights into immunotherapy for various conditions, ranging from immune disorders, metabolic ailments, and neural diseases to cancer. In the context of the retina, the activation of the cGAS-STING pathway has been linked to inflammation and degeneration [7]. The inhibition of cGAS-STING pathway alleviates neuroinflammation-induced retinal ganglion cell death after ischemia/reperfusion injury [8]. The cGAS-STING pathway contributes to the development of RPE senescence [9]. Ma et al. reported that PPARα could regulate cGAS-STING pathway activation in the oxygen-induced retinopathy (OIR) model [10], highlighting its potential impact on inflammation-associated angiogenesis in the retina. However, the specific mechanisms regarding to the downstream of cGAS-STING pathway and the intervention strategies within the eye remain elusive.

In this study, our analysis of a published retinal RNA-seq dataset revealed significant upregulation of *cGAS* and *STING* expression in patients with proliferative diabetic retinopathy (PDR). Further investigations in two well-established models of retinal/choroidal angiogenesis in *Sting* deficiency mice, laser-induced choroidal neovascularization (CNV) and OIR, demonstrated the crucial role of STING activity in retinal/choroidal angiogenesis. Targeting cGAS-STING pathway may be a novel immunotherapy approach for neovascular ocular diseases, and combination of STING inhibition and anti-VEGF drugs holds better treatment prospects.

## Methods

### Patients’ dataset and data analysis

A published RNA-seq data (GSE160306) of retinal tissues from healthy controls, diabetic patients, patients with non-proliferative diabetic retinopathy (NPDR), and PDR patients was downloaded and re-analyzed for screening the regulatory factors involved [11]. The data of macular and peripheral retinal tissues were used for analysis. DESeq2 (v1.30.1) was applied for identification of differentially expressed genes (DEGs) with the criteria of absolute fold change > 2 and adjusted P-value < 0.05. All the DEGs were used to conduct Gene Ontology (GO) enrichment analysis by clusterProfiler (v4.0.2). GO terms with adjusted P-value < 0.05 were considered significantly. Gene set enrichment analysis (GSEA) was conducted using the GSEA software (http://www.gsea-msigdb.org/gsea/index.jsp) with 1000 times gene-set permutations and cutoff values of FDR < 0.25. A power analysis was performed and statistical analysis was conducted using one-way ANOVA with Turkey’s post hoc test for comparisons of these four groups. Pearson’s correlation test was employed for correlation analysis between the expression of STING and the average expression from GO terms.

### CNV and OIR mouse models

All animal experiments were approved by Institutional Animal Care and Use Committee of Zhongshan Ophthalmic Center, Sun Yat-sen University (Ethic ID: 2020 − 102). The *Sting*^*gt*^ (#017537) mice were obtained from Jackson Laboratory and C57BL/6J mice were purchased from GemPharmatech Company. CNV model was established as previously described [12]. Briefly, laser photocoagulation was performed using an argon laser (Lumenis, Inc., Santa Clara, CA, USA) with a center wavelength of 532 nm, an incident power of 200mW, a spot size of 100 μm, and a pulse duration of 100ms to induce rupture of Bruch’s membrane. Intravitreal injections were performed using a 5-µL Hamilton syringe with a 33-gauge needle. A concentration gradient of STING inhibitors and activator was established to determine the optimal dosage. The following concentrations were set for the experiments: C-176 at 1 mM, 5 mM, and 25 mM; SN-011 at 0.8 mM, 4 mM, and 20 mM; and diABZI at 0.1 mM, 0.5 mM, and 2.5 mM. Fundus photography and fluorescein angiography by intraperitoneal injection of 2% fluorescein sodium solution (Alcon laboratories, TX, USA) (5 µl/g) were captured using the Micron IV retinal imaging system (Phoenix Research Laboratories, Pleasanton, CA, USA). OIR model was established as previously described [13]. These mice received a single intravitreal injection of STING inhibitors and activator at P12 and the extent of retinal angiogenesis was determined at P17.

### Cell enrichment of myeloid cells

Myeloid cells from retinae or choroidal-scleral complexes were isolated using the EasySep CD11b + Cell Isolation Kit (STEMCELL, Canada). Tissues (*n* = 5–7) were initially isolated on ice, mechanically fragmented, and digested in a solution containing 25 µl papain (5 µl/ml, Worthington) and 35 µl Dnase (7 µl/ml, Sigma-Aldich) at 35 °C for 8 min. The resulting single-cell suspension underwent EasySep Cell Isolation Kit protocol, including cell labeling, antibody incubation, magnetic bead binding, and cell separation. A purified CD11b + myeloid cell population (> 85% purity) was obtained for subsequent experiments (Fig. S1).

### Single-cell RNA sequencing data analysis

The scRNA-seq data for the retinae of control mice and OIR mice was retrieved from the GEO database (GSE152928) [13]. The filtering, clustering, and subclustering analysis were performed as described previously [13]. We further scored all subtypes of microglia using the irGSEA package (v1.1.2) for pathways such as ROS, IFN-α, Angiogenesis, and generated a graphical representation of the scores.

### Cell culture and treatment

Bv2, N9, Raw264.7 or bEnd.3 cells were cultured in DMEM/F12 containing 10% fetal bovine serum and 1% Penicillin-Streptomycin (Invitrogen). Hypoxic condition was produced in a Forma 3111 Series II Water Jacketed Incubator (Thermo Fisher Scientific; Waltham, MA, USA) with 5% CO2, 1.5% O2, and balance nitrogen gas. The cells were pre-stimulated with either C-176 (10µM) or vehicle before cultured in hypoxic condition for 24 h.

### Quantitative PCR analysis and western blotting

The qPCR was performed as previously described [14]. The 20 µl reaction mix included 2 µl cDNA, 10 µl 2×SYBR Premix Ex Taq, 7 µl ddH2O, and 10 µmol/l of primer pairs. Roche LightCycler480 II was used for qPCR, sequences of the primers used in this study were listed in the supplementary Table 1. The western blotting was performed as previously described [14], and the primary antibodies were listed in the supplementary Table 2.

### Immunofluorescence and hematoxylin & eosin staining

The immunofluorescence on whole-mounts and cryosections were carried out as previously described [13]. The primary antibodies were listed in the supplementary Table 2. The TUNEL assay was performed following the manufacturer’s protocol (Roche Diagnostics, Indianapolis, IN, USA). Confocal microscope images were captured using LSM980 (Carl Zeiss). H&E staining was conducted and the thickness of CNV lesions was measured as previously described [12, 13].

### Statistical analysis

The representative results were showed in the figures. Data quantification was presented as mean ± standard error of measurement (SEM). Statistical analysis was conducted using one-way ANOVA with Turkey’s post hoc test for comparisons of three or more groups, or a 2-tailed Student t-test for two-group comparisons. A p-value < 0.05 was considered statistically significant. (*p < 0.05; **p < 0.01; ***p < 0.001).

## Results

### cGAS-STING signaling involved in the development of PDR

**Fig. 1:**
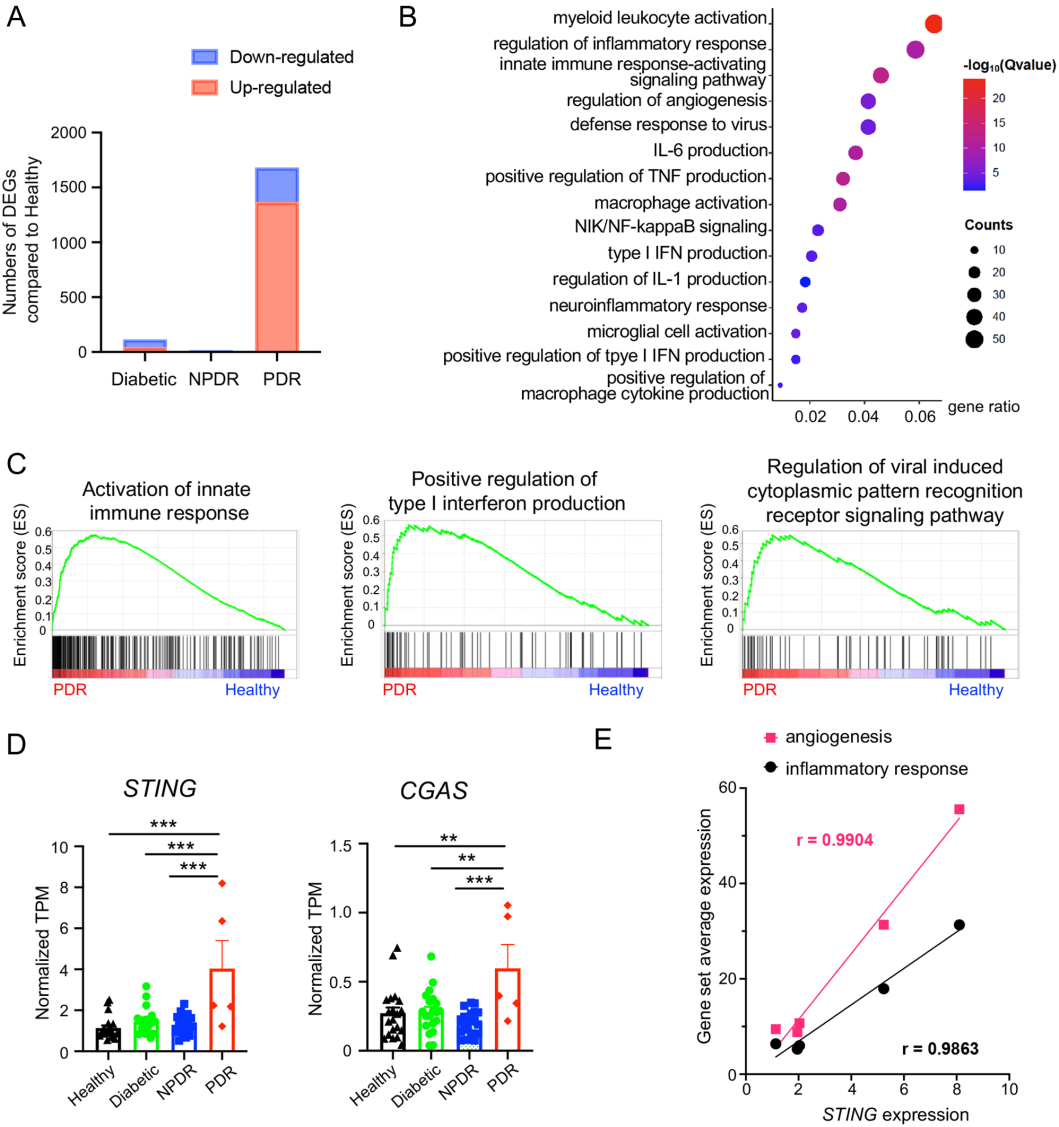
cGAS and STING was upregulated in the PDR patients. **A** The RNA-seq data was obtained from a published dataset (GSE160306) [11]. Compared to the Healthy control, there were 68 downregulated and 47 upregulated DEGs in the Diabetic group, 12 downregulated and 5 upregulated DEGs in the NPDR group, and 314 downregulated and 1369 upregulated DEGs in the PDR group. **B** Differentially expressed genes in retinae from PDR (*n* = 5 samples) and healthy control (*n* = 20 samples) were analyzed. The GO analysis of biological processes revealed that the differentially expressed genes in PDR retinae were enriched in myeloid leukocyte activation, regulation of inflammatory response, and other processes. **C** The GSEA profile showed that gene sets associated with the positive regulation of type I interferon production and the regulation of viral induced cytoplasmic pattern recognition receptor signaling pathway were upregulated in PDR retinae. **D** The expressions of *CGAS* and *STING* were markedly increased in the retinae from PDR patients (*n* = 5 samples) compared to healthy controls (*n* = 20 samples), diabetic (*n* = 20 samples) and NPDR patients (*n* = 19 samples). Power analysis was conducted to evaluate the reliability of detecting expression differences among the four groups, yielding a 99% power for *STING* and 96% power for *CGAS*. These high levels of statistical power provide confidence in the robustness of these comparisons using one-way ANVOA with Turkey’s post hoc test. **E** Linear regression analysis showed that the expression of *STING* was positively correlated with the up-regulated genes associated with angiogenesis and inflammatory responses in PDR patients (*n* = 5 samples). Data are shown as mean ± SEM. **P < 0.01; ***P < 0.001

To investigate the regulatory factors involved in the pathological process of retinal neovascularization, we downloaded and re-analyzed the published RNA-seq datasets of human retina [11]. Generally, the differentially expressed genes were enriched in the late DR stage of PDR but not other stages of diabetes and NPDR (Fig. 1A), which was coincident with original study by Beker et al. [11]. Specifically, we focused on the differentially expressed genes in the PDR stage. The GO analysis revealed that the upregulated genes in PDR mainly involved in the myeloid leukocyte activation, regulation of inflammatory response, innate immune response, type I interferon (IFN) production, and so on (Fig. 1B). In addition, GSEA analysis also showed genes related to positive regulation of type I IFN production and viral induced cytoplasmic pattern recognition receptor signaling pathway were up-regulated in the PDR patients compared with healthy controls (Fig. 1C). Furthermore, the expressions of *CGAS* and *STING*, key molecules of cytoplasmic DNA sensing pathway that induces the production of type I IFN and activates the innate immune system, were significantly elevated in PDR patients compared to others, whereas no significant difference was observed between diabetic or NPDR patients and healthy controls (Fig. 1D). Then the relationships between *STING* and the angiogenesis or inflammatory responses were analyzed. The expression of *STING* is positively correlated to genes associated with angiogenesis or inflammatory responses (Fig. 1E), indicating the possible involvement of STING signaling in the angiogenic process.

### cGAS-STING pathway was activated in two models of retinal/choroidal angiogenesis

**Fig. 2:**
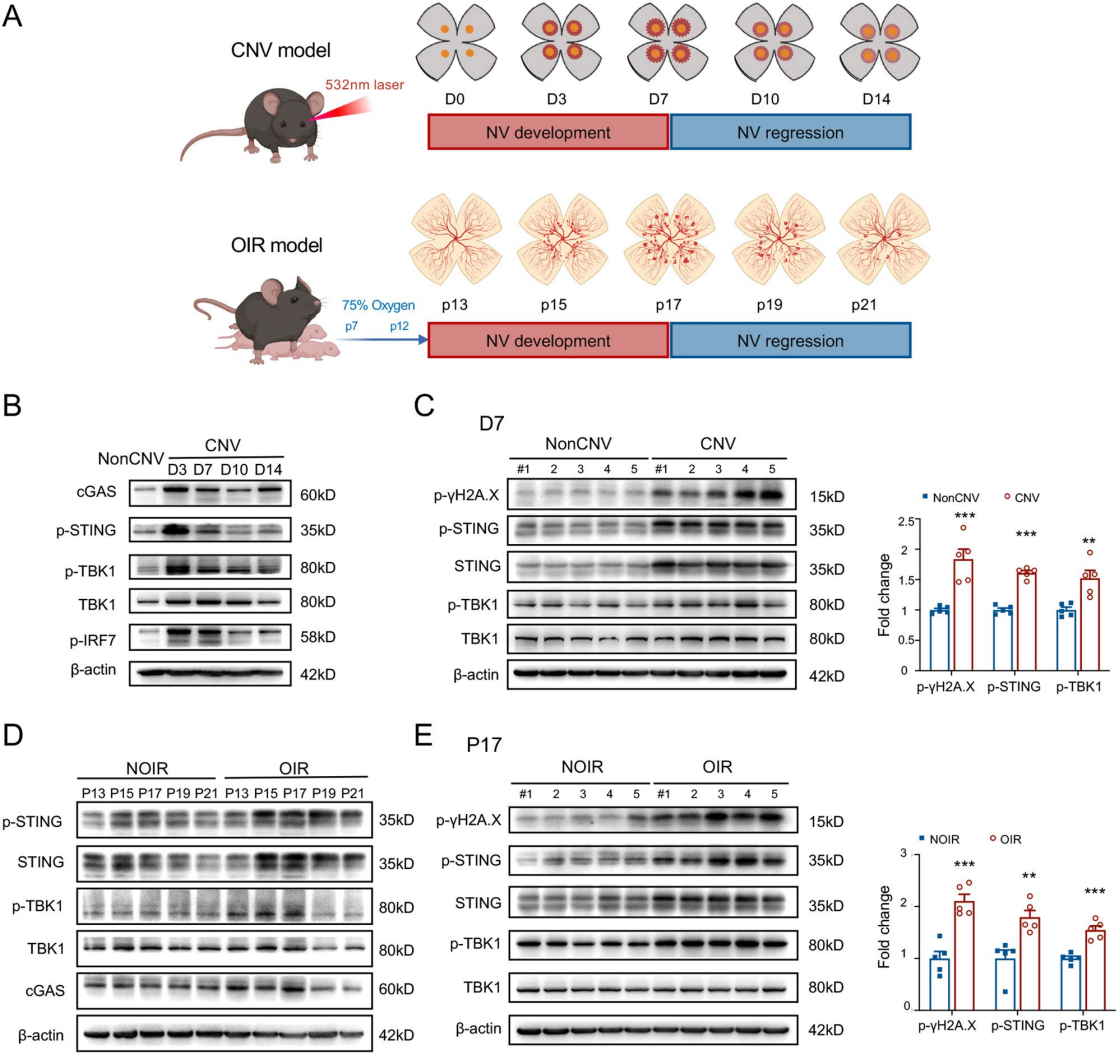
cGAS-STING pathway was significantly activated in CNV and OIR mice. **A** The schematic diagram illustrates the modeling process of CNV and OIR. **B** Western blot results display the expression changes of cGAS-STING pathway proteins, including cGAS, p-STING, TBK1 and p-TBK1 in CNV mouse choroids. The pathway proteins exhibit an initial increase, peak at D3, and gradually decrease thereafter. **C** The cGAS-STING pathway including p-γH2A.X, STING, TBK1 and their phosphorylation forms were detected on D7 (*n* = 5 choroid-sclera complexes). The statistical results were depicted. **D** The expressions of cGAS, STING, TBK1 and their phosphorylation forms were also examined in the OIR-retinae at various time points. **E** The cGAS-STING pathway was further detected on P17 (*n* = 5 retinae). The statistical results were depicted. D, day; P, postnatal day. Data are shown as mean ± SEM. *P < 0.05; **P < 0.01; ***P < 0.001

Next, we examined the cGAS-STING pathway in two classical models of retinal/choroidal angiogenesis, the CNV and OIR models (Fig. 2A). The laser-induced CNV is a self-limiting model of angiogenesis, with lesions peaking at D7 and then gradually regressing [15]. Another classical angiogenesis model, OIR, demonstrates the development of retinal neovascular tufts from P12 to P17, reaching their peak at P17, followed by a gradual regression of retinal angiogenic tufts [16]. As illustrated in Fig. 2B, the levels of cGAS and phosphorylated-STING (p-STING) in the choroid-scleral complex were notably up-regulated on D3 post laser administration and gradually declined thereafter, but still be elevated on D7. The downstream molecules, TBK1 and IRF7 also exhibited heightened activation on D3 and D7 (Fig. 2B). The expressions of p-γH2A.X, a marker of double-strand break (DSB) damage response signaling, p-STING, and p-TBK1 were significantly elevated on D3 and D7 post-CNV induction (Fig. 2C and S2A, B), indicating the involvement of the cGAS-STING axis in the induction of CNV. Similarly, the expressions of p-STING and p-TBK1 in OIR retinae were significantly increased as early as P13, with a prominent peak on P15, followed by a gradual decline (Fig. 2D). On P17, the peak time-point of retinal angiogenesis development in OIR, the cGAS-STING pathway was highly activated (Fig. 2E), indicating the contribution of the cGAS-STING pathway in the progression of pathological retinal angiogenesis. These findings collectively suggest that the activation of the cGAS-STING signaling contributes to the development of retinal/choroidal pathological angiogenesis in mice.

### Sting deficiency significantly alleviated the pathological angiogenesis

**Fig. 3:**
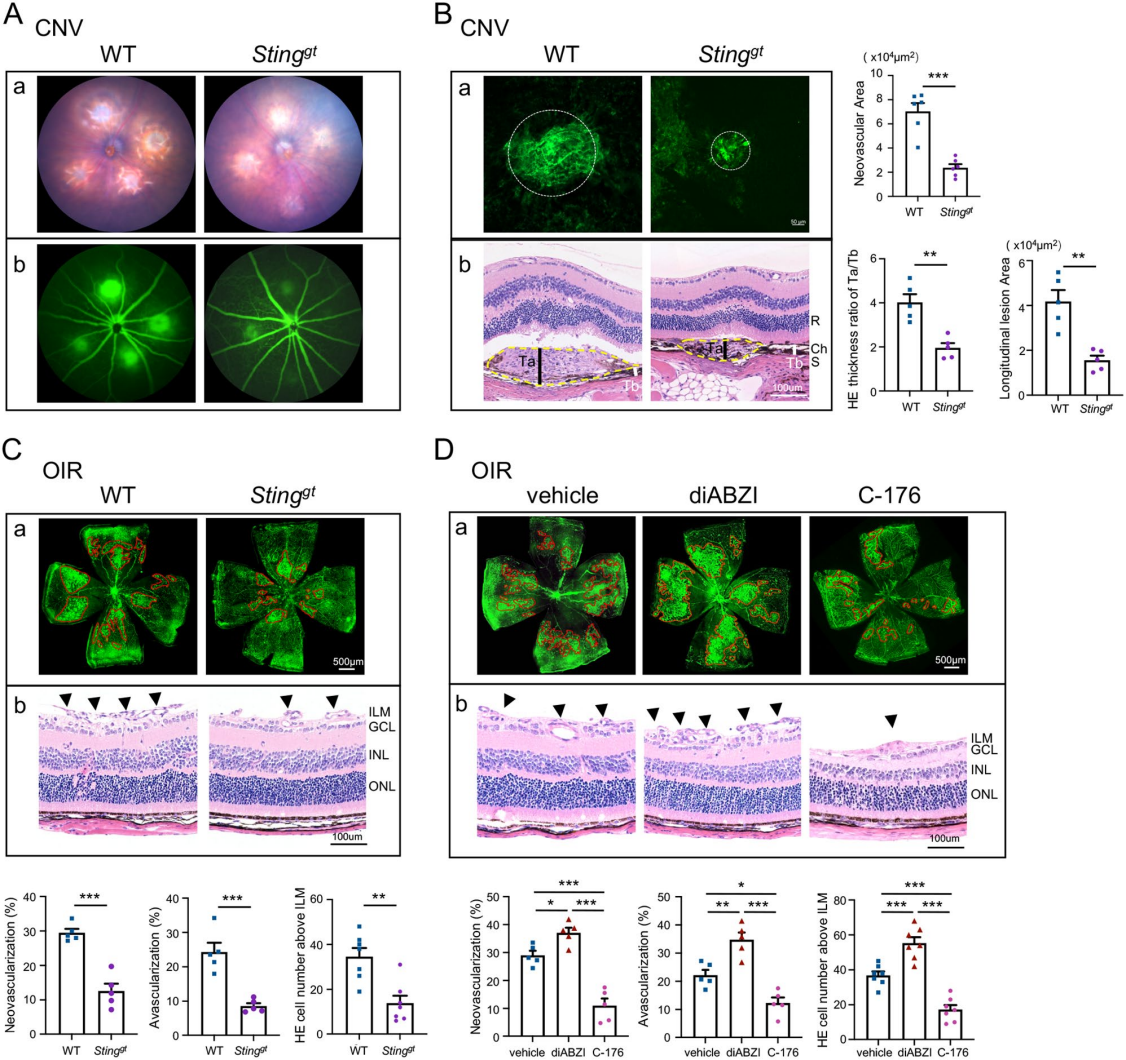
*Sting* deficiency suppressed the pathological angiogenesis in both the CNV and OIR mice. **A** Fundus photography (a) and fluorescein angiography (b) in the CNV models from WT and *Sting*^gt^ mice. The significantly alleviated laser burn lesions and reduced vascular leakage were observed in *Sting*^gt^-CNV mice. **B** CD31 staining on choroid-sclera complex flat-mounts showed that the CNV angiogenesis area (white circle) was much smaller in the *Sting*^*gt*^ CNV mice (a, *n* = 6 choroid-sclera complexes). HE staining on sections showing the CNV lesion areas delineated by the yellow dashed line (b, *n* = 5 eyes). It is quantified as Ta/Tb ratio and lesion area. Ta indicated the thickness of the lesion (black bar), while Tb indicated the thickness of adjacent normal choroid (white bar). **C** CD31 staining of retinal whole mounts (a, *n* = 5 retinae) and H&E staining (b, *n* = 7 eyes) in OIR mice and quantification results. *Sting*^*gt*^ mice significantly reduced the area of neovascular tufts compared with WT-OIR mice. **D** CD31 staining of retinal whole mounts (a, *n* = 5 retinae) and H&E staining (b, *n* = 7 eyes) in OIR mice and quantification results. Mice subjected to intravitreal injection of C-176 exhibited a reduction in neovascularization similar to *Sting*^*gt*^ mice, while mice injected with diABZI showed a significantly increased area of neovascularization compared to the vehicle controls. R, retina; Ch, choroid; S, sclera. ILM, internal limiting membrane; GCL, ganglion cell layer; INL, inner nuclear layer; ONL, outer nuclear layer. Scale bars were shown in the figures. Data are shown as mean ± SEM. *P < 0.05; **P < 0.01; ***P < 0.001

To further evaluate the effect of cGAS-STING pathway on the development of pathological angiogenesis, we established the CNV and OIR models utilizing *Sting*^*gt*^ mice, which harbor a mutant missense allele in the *Sting* gene resulting in the lack of STING protein [17]. As shown in the representative fundus images of Fig. 3A, laser burns were successfully induced in both WT and *Sting*^*gt*^ mice. The Bruch’s membrane was damaged by focal laser photocoagulation and the new formed and leaky choroidal blood vessels invaded into the retina in the CNV model [15]. When monitored by FFA, The *Sting*^*gt*^-CNV mice present relatively less leakage area (Fig. 3A). Choroid-sclera flat mounts and histological sections stained with HE also revealed that *Sting*^*gt*^-CNV mice displayed a smaller area of neovascularization and a thinner lesion thickness in both the transverse and longitudinal sections of the eyeball (Fig. 3B). Similarly, areas of retinal neovascular tufts and avascular zone were remarkably decreased in the *Sting*^*gt*^-OIR mice (Fig. 3C), and neovascular cells anterior to the internal limiting membrane (ILM), hallmarks of angiogenesis in OIR, were significantly reduced in the *Sting*^*gt*^-OIR mice (Fig. 3C). The pharmacological activation and inhibition of STING were also utilized to further evaluate the role of STING on retinal angiogenesis. We conducted dose-response experiments to determine the optimal dosage of C-176, SN-011, and diABZI. As shown in Fig. S3A-C, concentrations of 5 mM for C-176, 4 mM for SN-011, and 0.5 mM for diABZI were selected as the lowest effective concentrations for further study. Intravitreal injection of the STING inhibitor C-176 resulted in effects similar to the *Sting*^*gt*^ group, while administration of diABZI, a STING agonist that binds to the cGAMP binding pocket of STING, led to STING activation and larger area of neovascular tufts, indicating the essential role of STING activation for promoting pathological angiogenesis (Fig. 3D). In conclusion, these results demonstrated that the cGAS-STING signaling is required for the development of pathological angiogenesis.

### cGAS-STING signaling was required for myeloid cells activation during retinal/choroidal angiogenesis

**Fig. 4:**
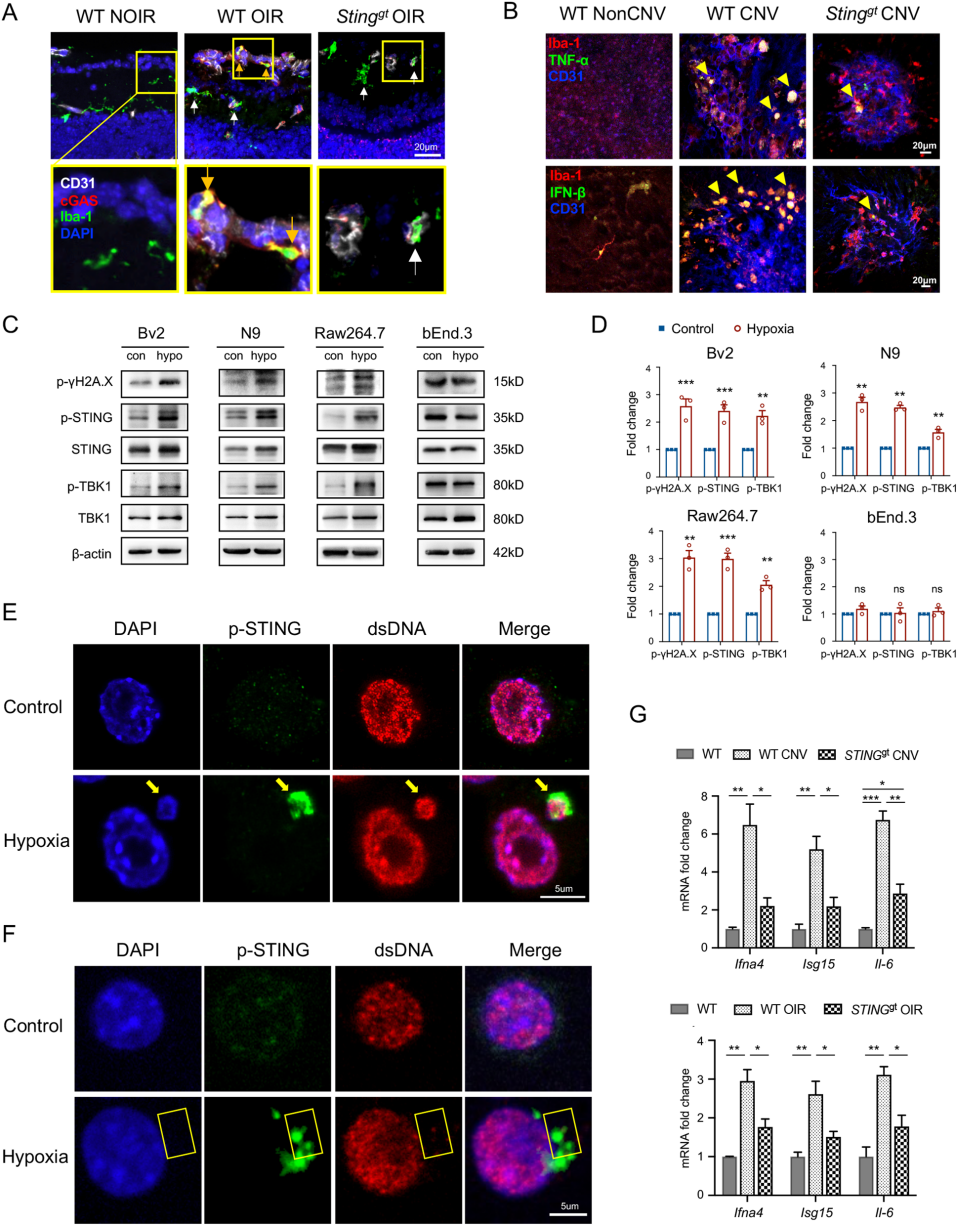
The cGAS-STING pathway was specifically activated in the myeloid cells. **A** In the cryosection immunofluorescence staining of WT-OIR mice, Iba-1 + myeloid cells adjacent to neovessels showed more co-localization with cGAS (yellow arrows), whereas Iba-1 + myeloid cells away from neovessels were lack of cGAS (white arrows). In addition, the *Sting*^*gt*^-OIR mice also showed less cGAS expression on the Iba-1 + myeloid cells. Yellow rectangles demonstrated the enlarged images. **B** Immunostaining of the choroid-sclera complex flat-mount revealed that WT-CNV mice exhibited more Iba-1 + myeloid cells infiltration, larger cell bodies, and a greater number of cells co-expressing TNF-α and IFN-β (yellow arrowheads). In contrast, *Sting*^*gt*^-CNV mice displayed a branched morphology similar to that of the controls and exhibited less co-localization with TNF-α and IFN-β. **C** and **D** In response to hypoxia stimulation, the expressions of p-γH2A.X, p-STING, and p-TBK1 were markedly elevated in Bv2 cells, N9 cells, and Raw264.7 cells, whereas these expression elevations were not observed in the bEnd.3 cells (*n* = 3 cultures). **E** and **F** Under hypoxia condition, cytosolic micronucleus with both DAPI and dsDNA stanning were co-localized with p-STING in N9 cells (E, yellow arrows). In addition, there were also some scattered cytosolic p-STING signaling co-localize with scattered cytoplasmic dsDNA signaling, indicating a possible leakage of mtDNA under hypoxic conditions. (F, yellow rectangles). Scale bars were shown in the figures. **G** qPCR results showed that compared to the WT-CNV and WT-OIR groups, Sting^gt^ groups exhibit lower expression of Il-6, Isg15, and Ifna4. (*n* = 3 eyes). Data are shown as mean ± SEM. *P < 0.05; **P < 0.01; ***P < 0.001; ns: no significance

To uncover the potential mechanisms underlying cGAS-STING activation during retinal/choroidal angiogenesis, we re-analyzed our previously published single-cell data in OIR on P17 [13]. Interestingly, the genes related to cGAS-STING signaling were enriched in the retinal myeloid cells (Fig. S4A), consistent with previous studies that STING is mainly expressed in myeloid cells located in central nervous system (CNS) [18]. Immunofluorescence staining of cryosection showed that the cGAS was specifically expressed on the Iba-1 + microglia that adjacent to neovascular tufts, while lack of cGAS expression was observed in the Iba-1 + microglia cells away from neovascular tufts, indicating the involvement of cGAS-STING signaling in the microglia activation and migration towards the neovascular tufts (Fig. 4A). The choroid-sclera flat-mounts staining showed that Iba-1 + myeloid cells around the vessels in WT-CNV mice exhibited the amoeboid activation morphology and expressed TNF-α and IFN-β, of which production were triggered by cGAS-STING signaling. By contrast, the *Sting*^*gt*^ mice presented much less TNF-α and IFN-β expressed on the Iba-1 + myeloid cells (Fig. 4B). Similarly, the *Sting*^*gt*^-OIR mice also exhibited less amoeboid Iba-1 + microglia around the CD31 + tufts, and the TNF-α and IFN-β expressed on the Iba-1 + microglia were significantly reduced (Fig. S4B) indicating the STING activation contributed to the microglia activation and the inflammatory responses during angiogenesis. In addition, the qPCR analysis demonstrated lower secretion of pro-angiogenic factors and reduced activation of interferon-stimulated genes (ISGs) in the *Sting*^*gt*^ group (Fig. 4G). Collectively, these results demonstrated STING activity in myeloid cells directed inflammatory responses and angiogenesis in the CNV and OIR models.

### Hypoxia-induced DNA damage and activation of cGAS-STING signaling in myeloid cells

Given that cytoplasmic DNA serves as a trigger for cGAS-STING signaling [19, 20], we aim to further investigate whether hypoxic stress induces cytoplasmic DNA leakage and downstream STING signaling activation in myeloid cells. Two microglia cell lines, Bv2 and N9, as well as a macrophage cell line, Raw264.7, were cultured under the hypoxic condition for 24 h. As expected, upregulation of p-γH2A.X was observed in all three cell lines in response to hypoxic insults (Fig. 4C, D). In addition, the cGAS-STING signaling was activated with up-regulation of cGAS, p-STING, and p-TBK1 in the hypoxia-stimulated myeloid cells. However, no such response was observed in the bEnd.3 endothelial cell line under hypoxic condition (Fig. 4C, D). Consistently, immunofluorescence staining revealed that hypoxia led to the accumulation of cytoplasmic DNA, possibly as the micronuclei from nuclear DNA leakage or mtDNA leakage from mitochondria (Fig. 4E, F and S5A-C). These cytoplasmic DNA were co-stained with p-STING, indicating the STING activation upon the recognition of cytosolic DNA in myeloid cells.

### cGAS-STING activation induced necroptosis in ocular myeloid cells

**Fig. 5:**
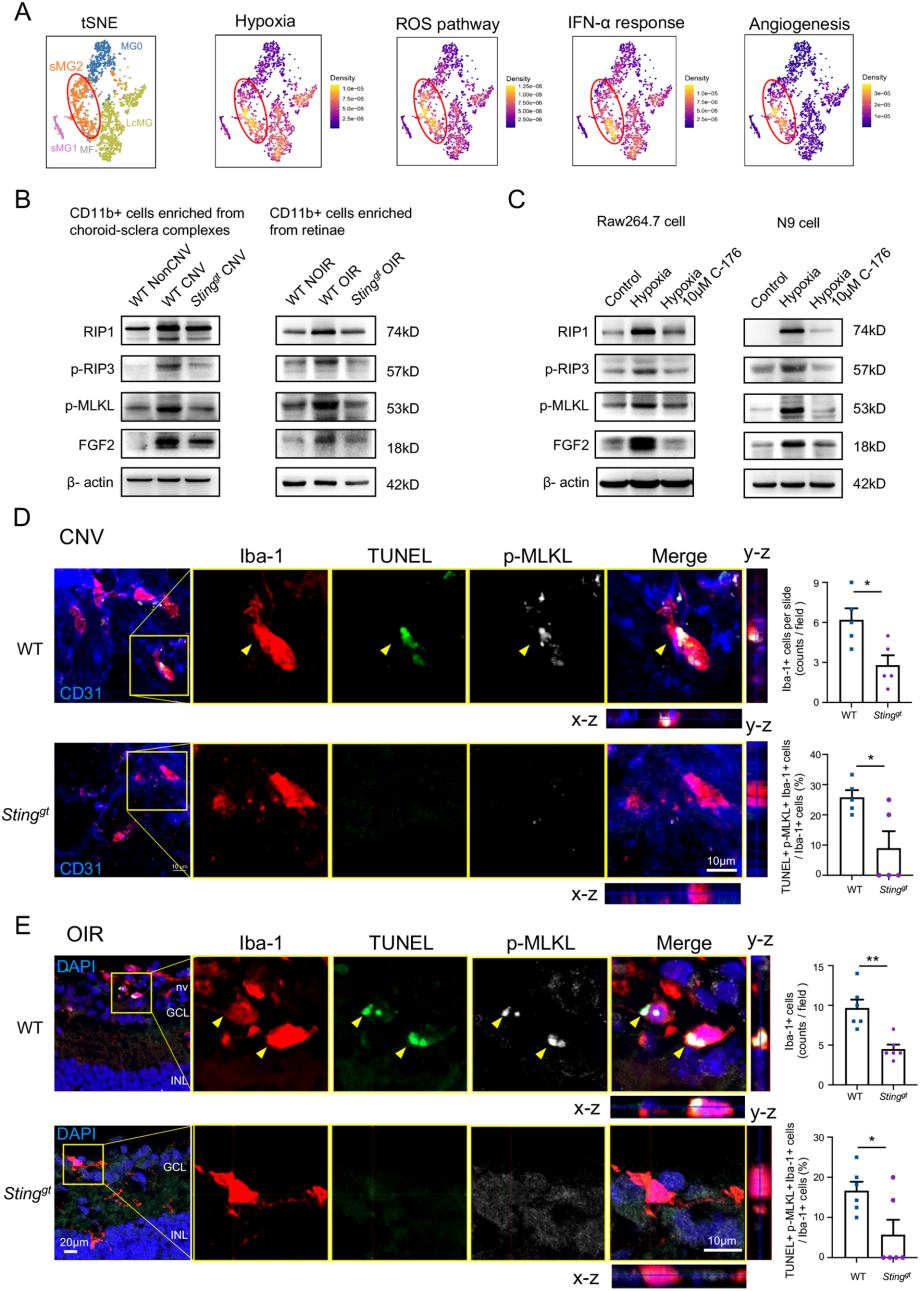
The cGAS-STING activation induces necroptosis in myeloid cells. **A** The single-cell data was obtained from a published dataset (GSE152928) [13].The tSNE plot showed five CD11b + clusters: MG0, homeostatic microglia; LcMG, a large cluster of microglia; MF, monocyte-derived and perivascular macrophages; and sMG1 and sMG2, two smaller clusters identified under hypoxic conditions. The irGSEA analysis revealed that the sMG2 subpopulation (red circles), associated with necroptotic processes, exhibited higher scores in hypoxia, ROS pathway, type I interferon, and angiogenesis biological processes, indicating the possible relation of necroptosis and cGAS-STING. **B** The expressions of necroptosis pathway molecules, including p-RIP3, p-MLKL, and FGF2 were detected in the enriched CD11b + myeloid cells from WT and *Sting*^*gt*^ choroid-sclera complexes on CNV-D7, as well as in the enriched CD11b + myeloid cells from WT and *Sting*^*gt*^ retinae on OIR-P17. **C** The expressions of necroptosis pathway molecules in Raw264.7 and N9 cell lines. **D, E** The TUNEL + p-MLKL + Iba-1 + necroptotic myeloid cells (yellow arrowheads) were significantly reduced in the CNV (*n* = 5 choroid-sclera complexes) and OIR (*n* = 6 eyes) from *Sting*^*gt*^ mice and the statistical results were depicted. nv, new vessels; GCL, ganglion cell layer; INL, inner nuclear layer. Scale bars were shown in the figures. Data are shown as mean ± SEM. *P < 0.05; **P < 0.01

Activation of cGAS-STING pathway leads to increased expression of downstream inflammatory factors, including I-IFN and TNF-α, which serves as a classical inducer of cell necroptosis. Our previous studies have found that microglia promoted the retinal angiogenesis through RIP3-mediated necroptosis [13]. Here, we hypothesize that the cGAS-STING signaling in ocular myeloid cells would induce their necroptosis, resulting in the development of angiogenesis. To validate this hypothesis, we firstly performed the GSEA analysis on the scRNA-seq data of the CD11b + cells. Among the five different CD11b clusters (Fig. 5A), sMG2 (small microglia cluster 2), a previously identified subset associated with necroptosis [13], was highly responded to hypoxia and exhibited increased IFN-α responses (Fig. 5A), indicating the involvement of cGAS-STING signaling in myeloid cells necroptosis. Furthermore, the expression of key proteins involved in the necroptosis pathway was examined in enriched CD11b + myeloid cells from both the CNV and OIR models. As expected, the p-RIP3, p-MLKL, and FGF2 were highly expressed in the myeloid cells enriched from WT-CNV and WT-OIR, which were abrogated by *Sting* deficiency (Fig. 5B), indicating the requirement of STING activity for necroptosis. Immunofluorescence staining and 3D co-localization analysis also showed that the Iba-1 + myeloid cells in *Sting*^*gt*^-CNV and *Sting*^*gt*^-OIR mice presented with less p-MLKL expression and reduced TUNEL positivity in comparison to WT groups (Fig. 5D, E). In vitro, hypoxia induced necroptosis with high expression of RIP1, p-RIP3, and p-MLKL in N9 and Raw264.7, which was suppressed by STING inhibitor of C-176, indicating the essential role of STING activation on necroptosis in myeloid cells (Fig. 5C). Taken together, these findings suggested that activation of cGAS-STING triggered necroptosis in myeloid cells, resulting in the development of pathological neovascularization.

### Enhanced inhibition of retinal/choroidal angiogenesis by combination treatment with anti-STING and anti-VEGF agents

**Fig. 6:**
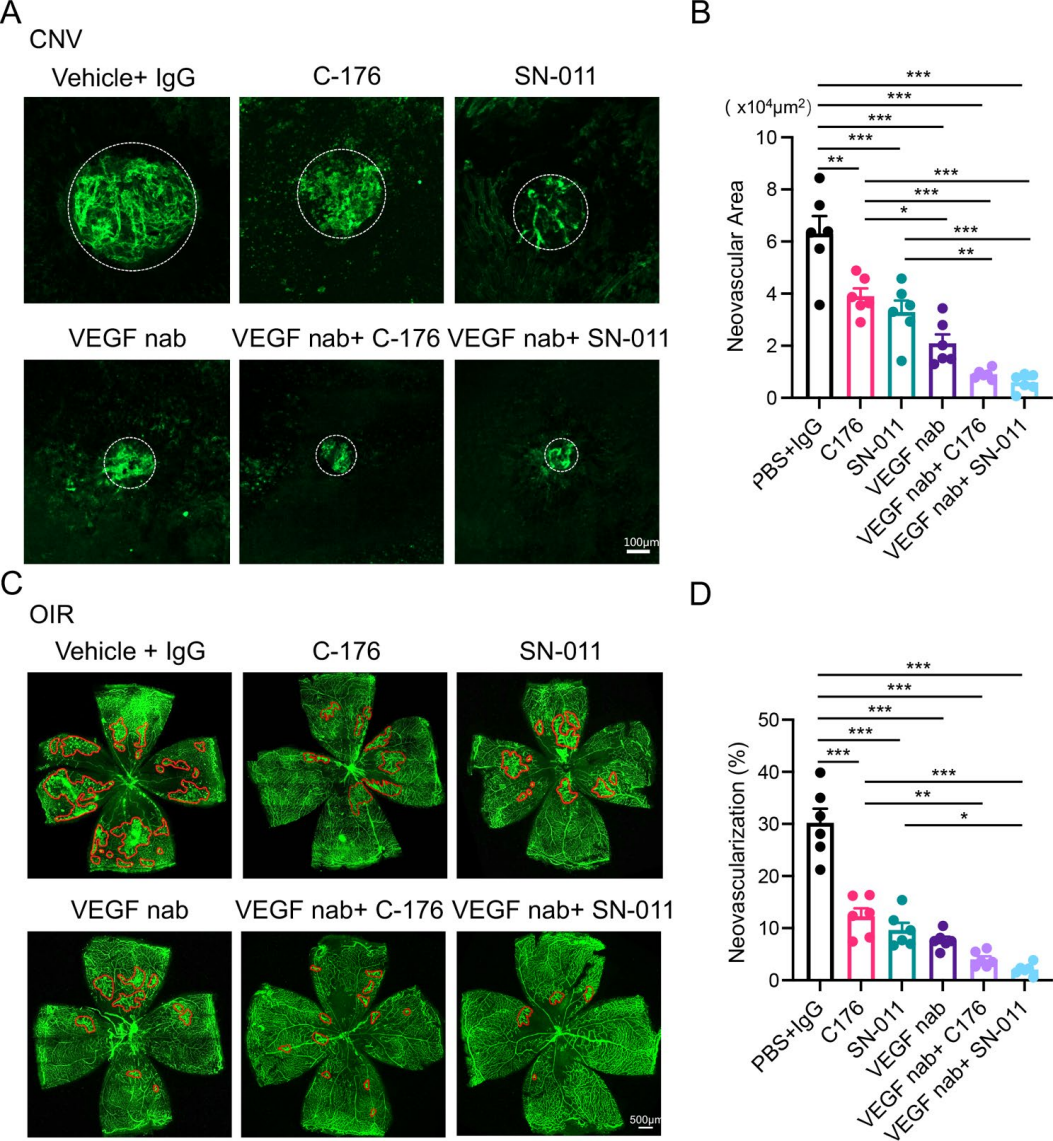
Combination therapy of STING inhibitors and Anti-VEGF agents in CNV and OIR Models. **A** Single intravitreal injection of inhibitors for STING (C-176 or SN-011) resulted in a notable reduction in angiogenesis development in CNV. The choroid-sclera complex flat-mounts displayed minimal angiogenic lesions following combined treatment with C-176 or SN-011 and anti-VEGF Nab. The statistical results are shown in **B** (*n* = 6 choroid-sclera complexes). **C** Single intravitreal injection of C-176 or SN-011 effectively suppressed angiogenesis in OIR. Notably, OIR retinae exhibited reduced angiogenic tufts following combined treatment with C-176 or SN-011 and anti-VEGF Nab. The statistical results were depicted in **D** (*n* = 6 retinae). Scale bars were shown in the figures. Data are shown as mean ± SEM. *P < 0.05; **P < 0.01; ***P < 0.001

Finally, we explored the therapeutic potential of targeting cGAS-STING signaling in neovascular ocular diseases. Two STING inhibitors, C-176 and SN-011 [21, 22], were employed to block the cGAS-STING axis through intravitreal injections in OIR and CNV mice. Both C-176 and SN-011 showed remarkable suppression of pathological vascular lesions, along with reduced FFA leakage areas in the CNV model (Fig. 6A, B). Moreover, combination therapy of C-176 or SN-011 with anti-VEGF neutralizing antibody resulted in the least leakage area (Fig. 6A, B), further highlighting the therapeutic potential of targeting the cGAS-STING axis in neovascular ocular diseases. Similarly, the intravitreal injection of C-176 or SN-011 could significantly inhibit the neovascular tufts areas and their combination therapy with anti-VEGF neutralizing antibody also present with least neovascular tufts areas in the OIR model (Fig. 6C, D). Collectively, our findings demonstrated that targeting STING by C-176 or SN-011 could effectively suppress retinal/choroidal pathological angiogenesis, and combination therapy with anti-VEGF could achieve enhanced efficacy.

## Discussion

**Fig. 7:**
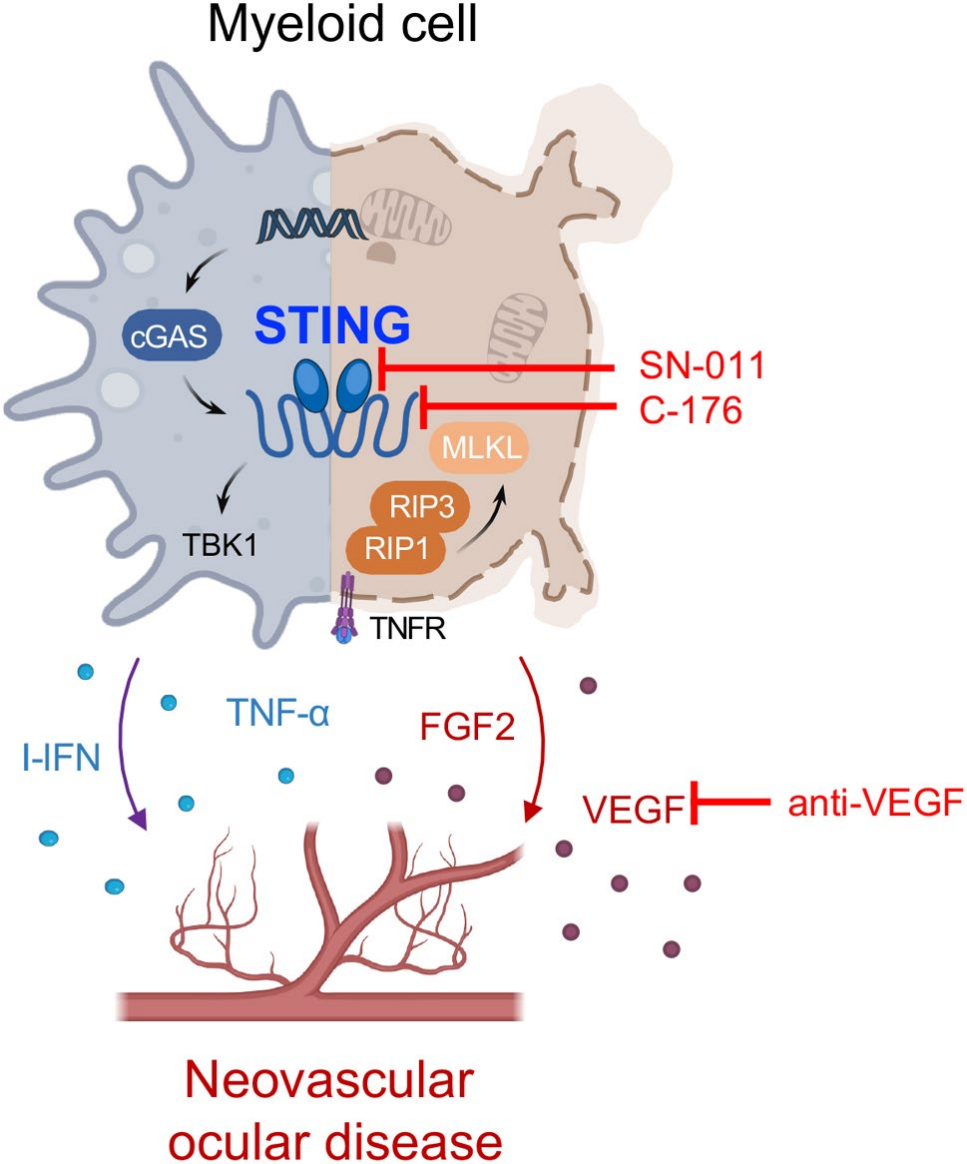
Schematic diagram. The activation of the cGAS-STING pathway in myeloid cells leads to necroptosis and subsequent pathological angiogenesis in neovascular ocular diseases. Under hypoxic conditions, the damaged double-stranded DNA triggers the activation of cytosolic cGAS-STING and release of I-IFN, TNF-α, and other inflammatory factors. In addition, the cGAS-STING activation upregulates the expression of necroptotic proteins, including RIP1, RIP3, and MLKL, leading to the secretion of pro-angiogenic factors such as FGF2 and VEGF. These events collectively promote the development of neovascular ocular diseases. Employing immunotherapy via SN-011 or C-176, which target distinct sites of the STING molecule, combined with anti-VEGF treatment, could effectively modulate the immune microenvironment for angiogenesis and suppress pathological neovascularization. Some icons were sourced from BioRender.com

Increasing evidence suggests that immune responses and inflammation play a crucial role in exacerbating neovascular ocular diseases [23]. Intraocular injections of steroids like dexamethasone and triamcinolone acetonide have shown promising effects in alleviating macular edema associated with various neovascular ocular diseases [3]. However, their non-specific property brings about side effects such as elevated intraocular pressure and cataract formation [3]. Understanding specific immune signaling pathways that mediate angiogenesis formation holds significant promise in exploring targeted immunotherapy for intervening in neovascular ocular diseases. Our study reveals the pivotal role of cGAS-STING signaling in promoting pathological angiogenesis through inducing necroptosis in myeloid cells. STING inhibitors, SN-011 or C-176, effectively mitigated retinal/choroid inflammation and neovascularization, indicating targeting cGAS-STING signaling may be a promising avenue for novel immunotherapeutic interventions in the management of neovascular ocular diseases (Fig. 7).

The emerging approach of immunotherapy has demonstrated considerable potential in the treatment of various diseases, including tumors and autoimmune conditions [24, 25]. By either stimulating or suppressing the body’s immune response, it educates the immune system to protect itself and prevent damage. In this study, we observed STING activation in retinal Iba-1 + microglia and choroidal Iba-1 + macrophages contributed to the pathological angiogenesis. Targeting the abnormally activated STING in myeloid cells could educate the immune system to stop the inflammation-related angiogenesis process, presenting as a novel immunotherapy approach. As we known, within the CNS, myeloid cells collectively refer to microglia, other CNS-associated macrophages, and monocytes due to their closely related cell types [26]. Here microglia are considered to be the predominant immune cells in the retinal neovascular environment, while monocyte-derived macrophages are the main immune cells in the choroidal neovascular condition. Our results demonstrated that the selective activation of STING in retinal microglia and choroidal macrophages emerge as a distinctive therapeutic target.

Our findings revealed that *STING*^*gt*^ mice were resistant to hypoxia-induced necroptosis, highlighting the essential role of STING signaling in triggering microglial necroptosis. In fact, the STING pathway has a multifaceted involvement in the regulation of diverse cell death mechanisms, including apoptosis, necroptosis, pyroptosis, and PANoptosis [27, 28]. For instance, administration of the STING agonist diABZI in the respiratory tract of mice triggers PANcroptosis in respiratory epithelial cells, resulting in the establishment of an ARDS model [29]. Here our results demonstrated that aberrant activation of STING in myeloid cells increased necroptosis events and subsequently resulted in pathological angiogenesis. Elucidating the intricate interplay between STING and necroptosis holds potential for uncovering innovative therapeutic strategies for angiogenic diseases. Interestingly, the *Sting* deficiency induce a reduction in cGAS expression in Fig. 4A, indicating the presence of a potential feedback loop between cGAS and STING. Previous studies have shown that activation of cGAS-STING pathway induces type I IFN expression, which in turn triggers the transcriptional activation of a series of downstream IFN-stimulated genes [27]. The promoter region of the cGAS gene contains two IFN-sensitive response elements that can potentially induce cGAS synthesis in response to type I IFN [30, 31]. Therefore, upon activation of STING by cGAS, downstream genes could subsequently activate cGAS, forming a feedback loop. Further research into regulation of cGAS-STING would elucidate the intricate relationship between these two molecules.

Hypoxia-induced VEGF expression and release have been considered to be the key mechanism mediating retinal/choroidal angiogenesis. Actually, anti-VEGF drugs have been widely used in the clinical treatment of neovascular ocular diseases such as PDR, wet AMD, etc. However, their efficacy is limited, and long-term sustainability still faces challenges. Other factors that mediate neovascularization need to be further considered. In this study, we found STING-activated myeloid cells orchestrated the pro-inflammatory microenvironment that favors an increased rate of angiogenesis. Our prior investigation has unveiled necroptosis-mediated FGF2 release from myeloid cells, suggesting a synergistic promotion of ocular pathological angiogenesis with VEGF [13]. This indicates that STING-necroptosis may function as an independent pathway, complementing the VEGF-mediated angiogenesis. Based on this background, the combination of immunotherapy targeting STING signaling and anti-VEGF agents was attempted. The results showed that this combination therapy could enhance the anti-angiogenic efficacy, indicating a promising clinical therapy for neovascular ocular diseases.

In this study, C-176 and SN-011, two small-molecule compounds of STING inhibitors were examined for treating pathological angiogenesis. As we known, STING inhibitors have seen application in diverse animal disease models, including systemic lupus erythematosus (SLE) [32], Parkinson’s disease [33], and ischemic stroke [34]. Notably, a recent clinical trial has been conducted for using the first inhibitor targeting the cGAS-STING pathway in SLE [35]. Here we found STING inhibitors presented promising candidates for neovascular ocular diseases. STING inhibitors typically falling into two classes: those binding to cysteine residues C88 or C91 near STING’s transmembrane domain, exemplified by C-176 and H-151 [36], and those occupying the CDN-binding site, acting as competitive antagonists of STING activators, including SN-011 and Astin C [22, 37]. Here we found both types of inhibitors achieved similar degree of angiogenesis suppression, but C-176 required a higher concentration (Fig. 6A, C). Studies have shown that C-176 and its analogs are Cys-reactive drugs, known for nonspecific binding to unpaired Cys in various proteins, suggesting more off-target activities than SN-011 [21]. Collectively, we proposed that SN-011 might offer better selectivity and safety for suppressing angiogenesis. Moreover, unlike systemic administration in previous studies, the intravitreal injection method in this study allows for lower drug doses, minimizing the potential systemic risk [37].

## Conclusion

In summary, our findings demonstrated the activation of the cGAS-STING pathway in retinal microglia and choroidal macrophage facilitated the process of RIP3-MLKL-mediated necroptosis, ultimately leading to neuroinflammation and pathological angiogenesis. Targeted inhibition of the cGAS-STING pathway with C-176 or SN-011 could significantly suppress the myeloid cell necroptosis-mediated neuroinflammation and pathological angiogenesis. Additionally, combination of STING inhibitors, SN-011 and C-176, with anti-VEGF agents exhibited enhanced anti-angiogenic efficacy. These findings pivotally position the cGAS-STING-necroptosis axis as a novel and promising target for immunotherapeutic intervention.

### Electronic supplementary material

Below is the link to the electronic supplementary material.

Supplementary Material 1

Supplementary Material 2

Supplementary Material 3

## Data Availability

A published RNA-seq data (GSE160306) of retinal tissues was downloaded and re-analyzed.
