# Supplementary material for: Therapeutic intervention in neuroinflammation for neovascular ocular diseases through targeting the cGAS-STING-necroptosis pathway
Source: J Neuroinflammation. 2024 Jun 25;21:164. doi: 10.1186/s12974-024-03155-y (PMC11197344; doi:10.1186/s12974-024-03155-y)

Supplementary Fig. 1 Flow cytometry analysis of enriched CD11b + myeloid cells.

Flow cytometry was utilized to evaluate the purity of CD11b + myeloid cells isolated using a specific enrichment kit. The analysis revealed that 87.0% of the gated cell population positively expressed CD11b, confirming the high purity and successful enrichment of the targeted myeloid cells.


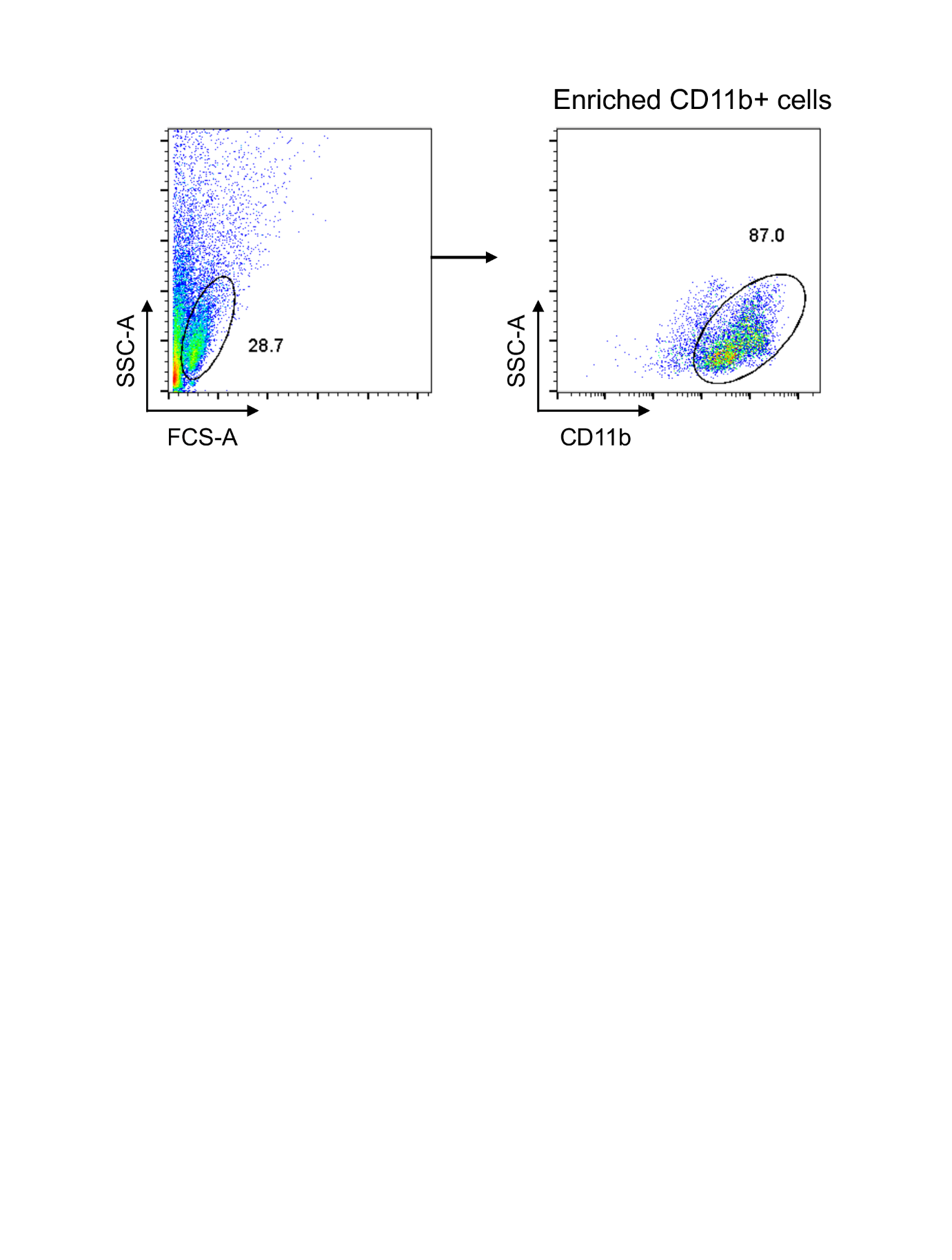


Supplementary Fig. 2 Expression of cGAS-STING pathway components on Day3 in CNV model. **A** The cGAS-STING pathway including p-γH2A.X, STING, TBK1 and their phosphorylation forms were detected on D3 (n = 4 choroid-sclera complexes), and the expression of p-γH2A.X, p-STING, and p-TBK1 were significantly upregulated. The statistical results were presented in **B**. Data are shown as mean ± SEM. **P* < 0.05; ***P* < 0.01.


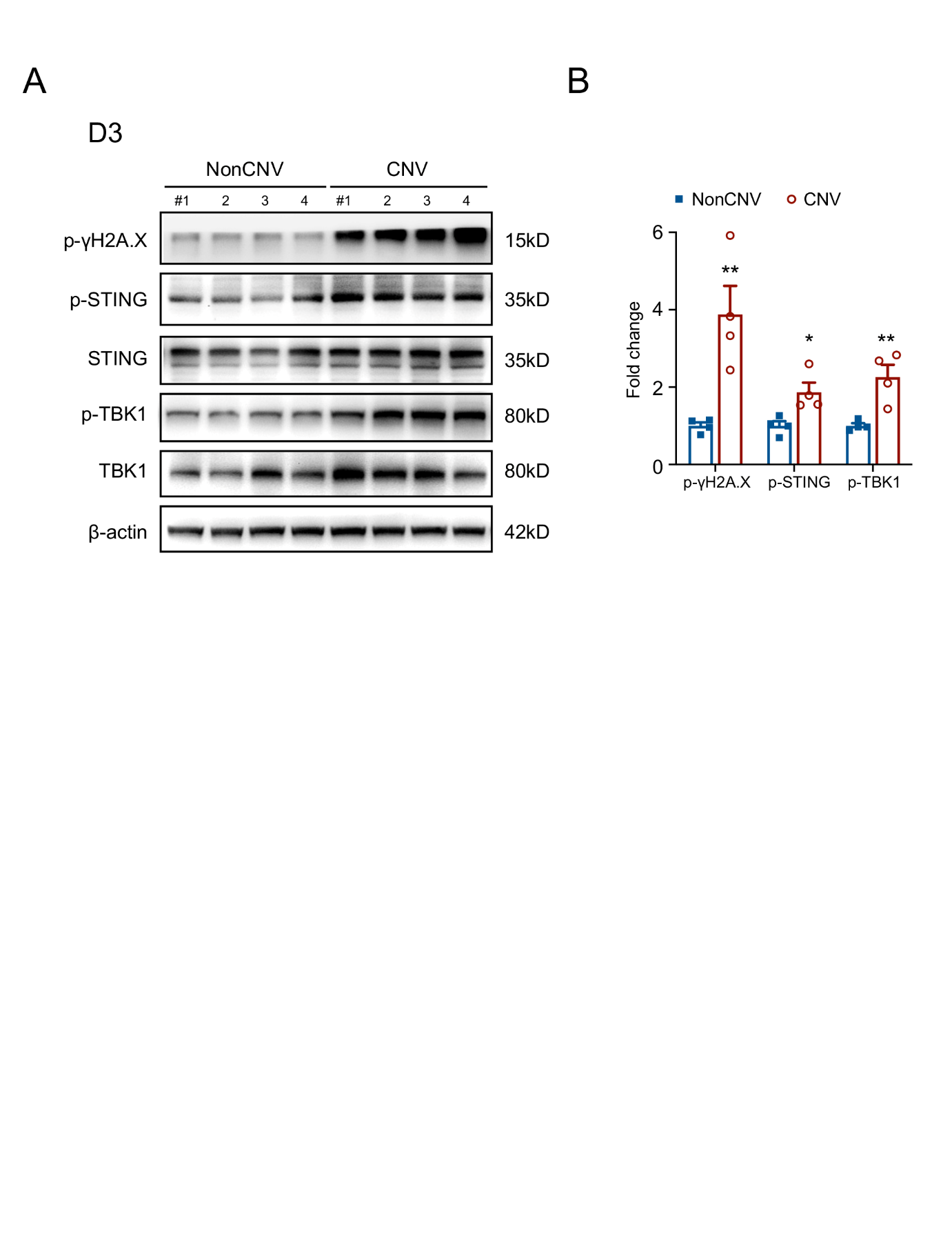


Supplementary Fig. 3 Dose-response experiment of C-176, SN-011, and diABZI in CNV model. **A** Representative fundus photography (a), fluorescein angiography (b), and CD31 staining on choroid-flat-mounts (c) show the effects of varying C-176 concentrations on CNV. Both 5 mM and 25 mM of C-176 significantly reduced neovascularization, demonstrating comparable effects. In contrast, 1 mM dose did not show any significant improvement compared to the control. The statistical results were depicted (n = 3 choroid-sclera complexes). **B** Images from fundus photography (a) fluorescein angiography (b) and CD31 staining on choroid-flat-mounts (c) for SN-011 treatments revealed that concentrations of 4 mM and 20 mM effectively inhibited neovascularization without significant differences between them. In contrast, the 0.8 mM dose had no significant impact. The statistical results were depicted (n = 3 choroid-sclera complexes). **C** For diABZI, fundus photography (a) fluorescein angiography (b) and CD31 staining on choroid-flat-mounts (c) demonstrated the increased neovascularization at 0.5 mM and 2.5 mM. However, the 2.5 mM dose caused hemorrhaging, marked by yellow arrowhead. The statistical results were depicted (n = 3 choroid-sclera complexes). Scale bars were shown in the figures. Data are shown as mean ± SEM. **P* < 0.05; ***P* < 0.01; ****P* < 0.001.


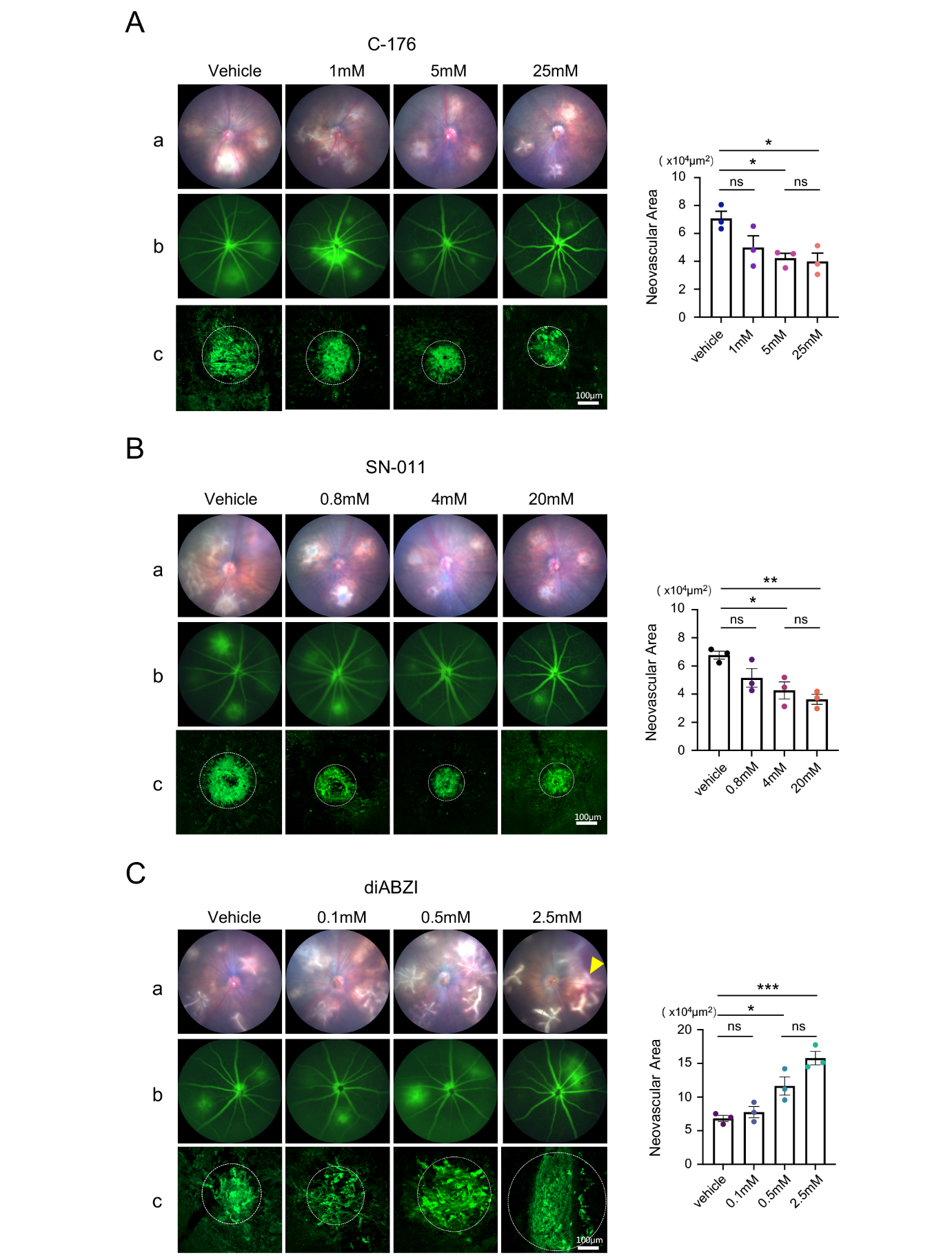


Supplementary Fig. 4 Expression of interferon stimulated genes and immunostaining in retinal flat-mounts. **A** Single-cell data analysis revealed that Tmem173 (Sting), Tbk1 and other interferon stimulated genes (ISGs) are predominantly expressed in myeloid cells. **B** Immunostaining of retinal flat-mount showed that WT-OIR mice exhibited more Iba-1 + myeloid cells infiltration, larger cell bodies, and more cells co-expressing TNF-α and IFN-β (yellow arrowheads). In contrast, Stinggt-OIR mice showed similar branched morphology to the WT-NOIR controls and less co-localization with TNF-α and IFN-β than that of WT-OIR mice.


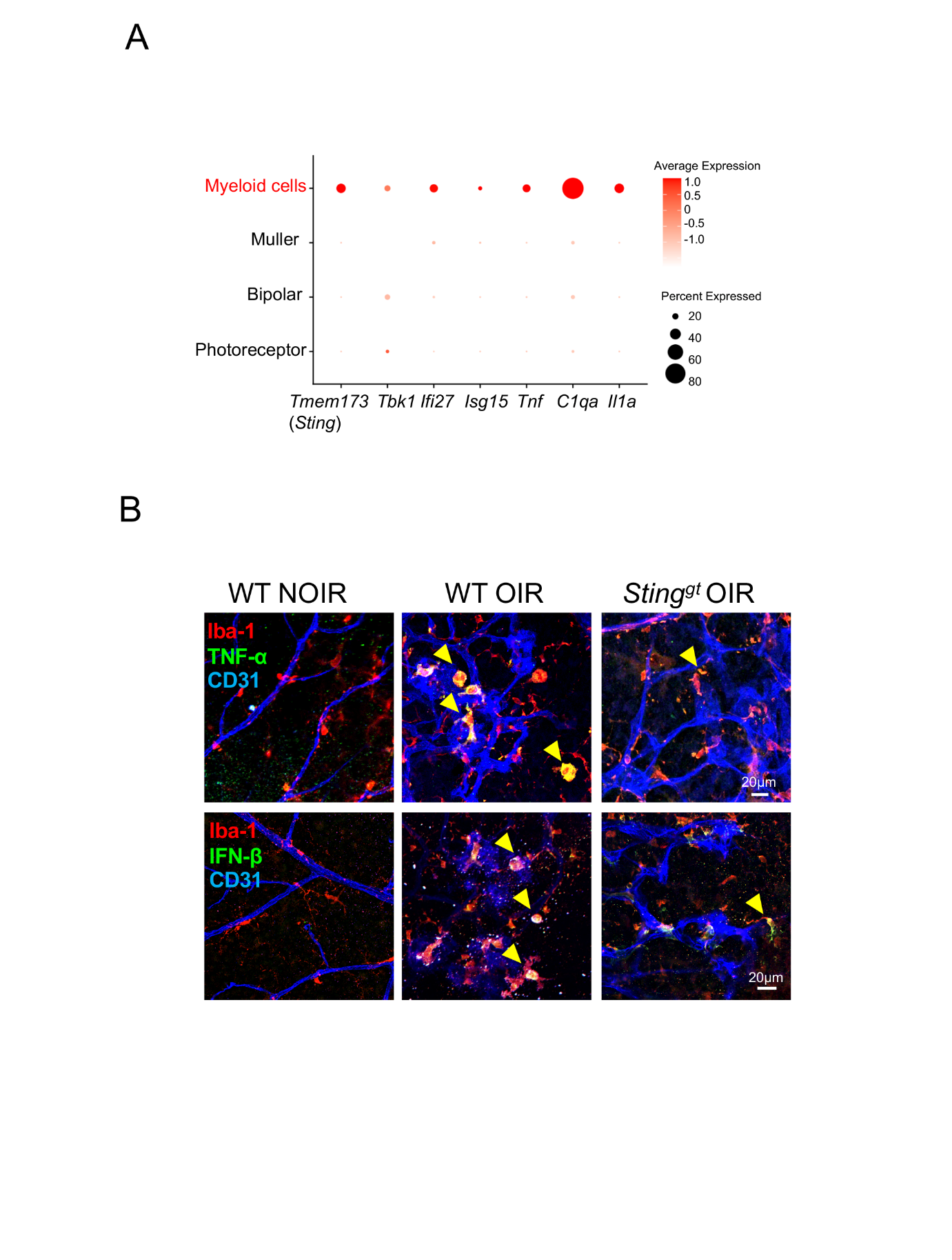


Supplementary Fig. 5 Hypoxia induced cytoplasmic DNA leakage and STING activation in myeloid cell lines. **A** Under hypoxic conditions, cytoplasmic micronuclei (DAPI + dsDNA+) in Bv2 cells were co-localized with p-γH2A.X signals, indicating hypoxia induced DNA damage and leakage into the cytoplasm. **B** The DAPI-dsDNA + cytoplasmic micronuclei was co-stained with p-STING in hypoxic Bv2 cell, indicating the cytoplasmic would trigger the STING activation. **C** RAW264.7 cells also displayed cytoplasmic micronuclei with colocalization of DAPI but not TOM20, as well as scattered p-STING signals within the cytoplasm under hypoxic condition. Scale bars were shown in the figures.


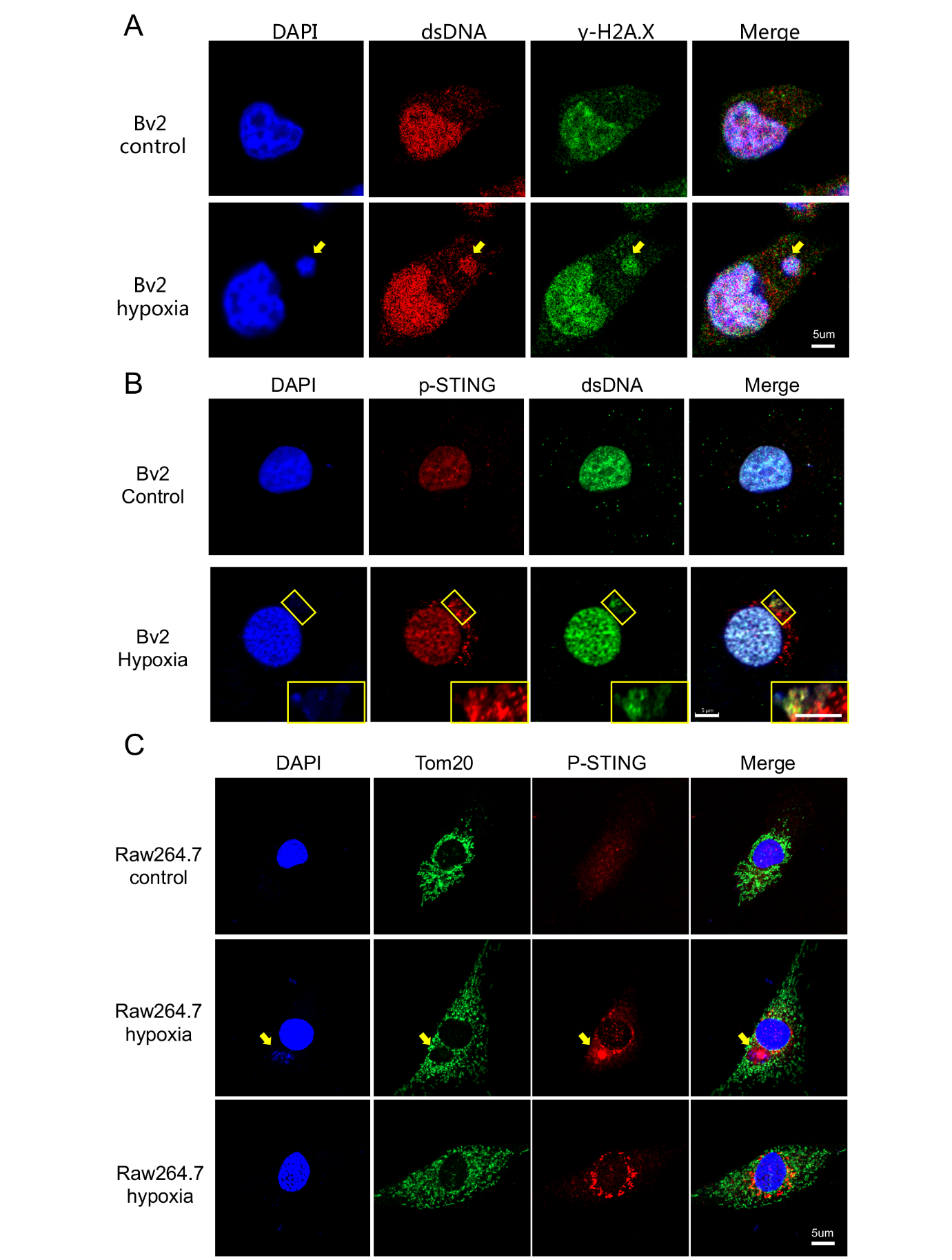

Supplement: Supplementary file 1 — Supplementary Material 1 [file 12974_2024_3155_MOESM1_ESM.docx]
